# Probing hybrid metallic sandwiches with nonlocal four-terminal electrical measurements

**DOI:** 10.1038/s41598-025-07507-3

**Published:** 2025-07-02

**Authors:** Mikhail Belogolovskii, Magdaléna Poláčková, Elena Zhitlukhina, Branislav Grančič, Leonid Satrapinskyy, Pavol Ďurina, Maroš Gregor, Tomáš Plecenik

**Affiliations:** 1 Kyiv Academic University, Academician Vernadsky Blvd. 36, Kyiv, 03142 Ukraine; 2 Centre for Nanotechnology and Advanced Materials, Faculty of Mathematics, Physics and Informatics, Mlynská dolina, Comenius University Bratislava, 84248 Bratislava, Slovak Republic; 3 O.O. Galkin Donetsk Institute for Physics and Engineering, National Academy of Sciences of Ukraine, Nauki Ave. 46, Kyiv, 03028 Ukraine

**Keywords:** Four-terminal electrical probing, Nonlocal measurements, Perpendicular four-probe resistance, Hybrid non-magnetic/ferromagnetic/non-magnetic metal trilayers, Nanoscience and technology, Physics

## Abstract

Despite its history of more than a century, the four-probe technique has remained a cornerstone of electrical measurements in thin conductive layers. Its traditional *on-sample* configuration consists of four electrical contacts arranged along a straight line to measure *local* sheet resistances. In multilayers, the results of such measurements cannot be interpreted straightforwardly due to their significant dependence on the properties of individual films and contact resistances between them. To address this challenge, we propose a *through-sample nonlocal* four-terminal method based on the Landauer-Büttiker scattering approach, which has been tested on hybrid all-metallic sandwiches composed of two 80 nm thick NbN films and a 50 nm thick core made of three archetypal ferromagnets, Co, Ni, or NiCu alloy. Results obtained for the trilayers are compared with the corresponding data for single NbN films 160 nm thick. At temperatures above the critical temperature of NbN, we have found negative values of nonlocal resistances which are explained using an equivalent circuit model with six resistances connecting the four probes. The key advantage of the proposed methodology lies in its simple design enabling the detection of subtle physical effects in transversely heterogeneous devices that might otherwise go unnoticed.

**Supplementary Information:**

The online version contains supplementary material available at 10.1038/s41598-025-07507-3.

## Introduction

Four-point electrical probing has become a major interdisciplinary technique used in applied and fundamental physics, semiconductor industry, geology, etc. to characterize solid materials by measuring their resistances^1^. This method using an *on-sample* setup with two outer contacts supplying and draining the current as well as the inner pair of contacts measuring the voltage drop *V* is applied primarily to determine the sheet resistance. The separation of current and voltage terminals eliminates the lead and contact influence on the *locally* probed resistances. Next, van der Pauw^2,3^ proposed an original procedure for determining specific electrical resistance (resistivity) *ρ* of the sample of arbitrary shape with four probes located on the specimen’s periphery. In such *nonlocal* experiments with two contacts A and B for the current *I*_AB_ and the other two C and D serving to measure the voltage drop *V*_CD_, the *V*_CD_ value is found in regions far from the nominal current path and is therefore controlled by the entire current distribution across the sample. The measured *nonlocal* resistance is determined as

1 $${R_{{\text{AB, CD}}}}={V_{{\text{CD}}}}/{I_{{\text{AB}}}}$$

The development of a three-dimensional heterogeneous integration technology based on the vertical stacking of thin films of different types^4,5^ requires a generalization of the four-terminal methodology to the case of currents flowing perpendicular to the planes (CPP) in the multilayers under study^6^. In particular, such samples include sandwiches consisting of non-magnetic (N) and ferromagnetic (F) metal films, for example, giant magnetoresistance devices^7,8^. If the electronic properties of the layers differ fundamentally, the presence of interfaces can lead to proximity effects with material properties “leaking” into each other and thereby creating new functionalities otherwise absent in isolated components^9^. One of the most interesting heterostructures in this regard are superconductor/ferromagnet/superconductor (S/F/S) trilayers that are at the forefront of proximitized materials research thanks to unique properties arising due to competing quantum effects of Cooper pairing and magnetic exchange interactions^10,11^. The product of their area and the normal CPP resistance *R*_n_, the main characteristic of metallic S/F/S junctions in the normal (n) state, is primarily determined by the charge transport across the S/F interface^12,13^. Therefore, its knowledge is very important for improving corresponding superconducting circuitries, while experimental definition and interpretation of the *R*_n_ value represent a much more complex task than a similar (already standard) problem for a parallel charge flow^14^. For this reason, the multilayer specimen has been often modeled as an equivalent circuit with several parallel resistances^6,15,16^. This approach allowed the authors to leave the already standard on-sample four-probe configuration unchanged, although ignoring the role of contact resistances between layers left unattended some physical phenomena in the studied hybrids, which are discussed below.

**Fig. 1:**
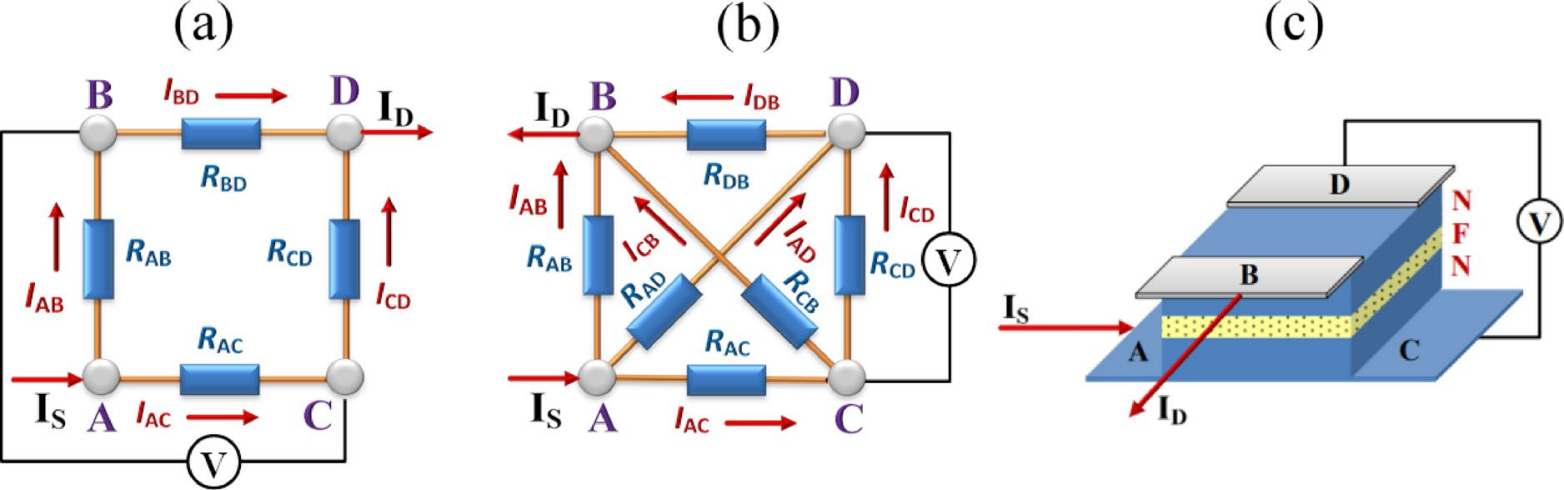
Four-probe non-local configurations for resistance measurements of a non-magnetic/ferromagnetic/non-magnetic (NFN) sandwich, when the СPP charge flow is driven from source *I*_S_ to drain *I*_D_ and the voltage drop is measured between two other nodes bounding the related conditional resistor(s). (**a**) Conventional four-resistor model^24–26^ with the crossed configuration of current and voltage contacts. (**b**) Circuit diagram with six conditional resistors for *R*_AB, CD_ measurements. (**c**) Schematics of the through-N/F/N sample four-terminal configuration with contacts B and D made of Pt, two NbN superconducting films with contacts A and C, and the ferromagnetic interlayer F in grey, blue, and yellow, respectively. The currents *I*_XY_ mean the net currents flowing from contact X to contact Y (X, Y = A, B, C, D and X *≠* Y), the arrows correspond to the expected directions of the charge flow and, accordingly, to positive current values. If, due to the charge transport redistribution, the current between the two nodes, by which the voltage drop is determined, changes its direction (and consequently the voltage drop changes its sign), the four-probe resistance calculated using Eq. (1) will be negative.

As for СPP measurements, one of the surprising discoveries was the detection, more than fifty years ago, of nonlocal negative resistance in mesoscopic sandwiches with two metal electrodes separated by an insulating layer^17^. Let us emphasize that it was not the negative *differential* resistance *dI*(*V*)/*dV* < 0, which appears in a limited voltage range away from *V* = 0 but rather the absolute negative value of the four-probe resistance determined by Eq. (1), which is undoubtedly *apparent* and emerges when the difference in electrical potentials between two voltage contacts is opposite to the expected one^18^. Following theoretical predictions^19,20^previously observed negative values of the four-terminal resistance^17,18,21–23^ have been obtained for the crossed configuration of current and voltage contacts shown in Fig. 1a. The explanation for the inequality *R*_AD, CB_ < 0 was based on the corresponding four-resistor model^24–26^ (Fig. 1a) or its more advanced finite-element modification^17,21^. This approach predicts positive *R*_AB, CD_ (Fig. S1a) while *R*_AD, CB_ (Figs. 1 and S1b) values can be negative, see the figures and related discussions in Supplementary Material.

The conclusion *R*_AB, CD_ > 0 contradicts experimental data for our three-dimensional version of the four-terminal configuration shown in Fig. 1c as well as the general theoretical approach to transport in mesoscopic systems, see below. To resolve the inconsistencies, we have revisited the four-resistor approximation used before^24–26^ and developed a simple heuristic model (Fig. 1b) that differs by introducing two additional scattering links. To experimentally reproduce its main features, a non-trivial way of connecting contacts to the sample under study was used, as well as its dimensions. Usually, the multilayer stack is patterned into pillars with designed dimensions of tens or hundreds of nanometers, see, e.g., the work^27^. After patterning, the pillars are passivated and a thick, highly conductive top electrode is applied to them, extending along the entire area of the pillar. This ensures the creation of an equipotential surface which largely eliminates the effects of unavoidable sample inhomogeneities^28^. On the contrary, our task was to create contact pads small compared to the sample size, the transverse dimensions of which were increased to 25 μm × 25 μm. The trilayers obtained were passivated with side bilayers formed by a 30 nm thick AlN layer and about 200 nm thick SiO_2_ oxide and two 35 nm thick platinum strips were deposited on top of them. A schematic view of the studied sandwiches and their biasing is shown in Fig. 1b, while Fig. S6 in Supplementary Material demonstrates the related cross-section. Using this approach, we found that the CPP resistance *R*_AB, CD_ (Fig. 1c) of the samples with the internal structure intentionally modified using various F interlayers, turned out to be very sensitive to small local fluctuations in the normal and superconducting parameters in depth.

## Results

### Six-resistor model for nonlocal four-terminal probing

In mesoscopic physics, the charge transport in a multi-terminal structure is usually described by the Landauer-Büttiker approach relating the electrical resistance to the scattering properties of the system^20,29^. In its linear-response version, the current leaving contact X is defined by the equation equivalent of related Ohm’s formula ${I_{\text{X}}}=(2{e^2}/h)\sum\limits_{j} ( {T_{{\text{XY}}}}{V_{\text{X}}} - {T_{{\text{YX}}}}{V_{\text{Y}}})$, where *T*_XY_ is the probability of charge transfer from terminal X to terminal Y, ${V_{\text{Y}}}={\mu _{\text{Y}}}/e$, *µ*_Y_ is the related chemical potential, X, Y = A, B, C, D, and X *≠* Y. The currents *I*_XY_ in Fig. 1 and S1 in Supplementary Material mean the net charge flows from contact X to contact Y: ${I_{{\text{XY}}}}=(2{e^2}/h)({T_{{\text{XY}}}}{V_{\text{X}}} - {T_{{\text{YX}}}}{V_{\text{Y}}})$. Leaving quantum analysis with related interference effects for the future, we restrict ourselves to the classical limit having the advantage to transform a material property directly into a qualitatively understandable concept. In the absence or presence of a relatively weak magnetic field, *T*_XY_ and *T*_YX_ probabilities coincide and the above expression for *I*_X_ will depend on the ${V_{\text{X}}} - {V_{\text{Y}}}$ difference. As a result, we can introduce “resistances” describing such contributions ${R_{{\text{XY}}}}=h/(2{e^2}{T_{{\text{XY}}}})$ and then proceed with the four-probe configuration as a circuit of six resistors by applying the known Kirchhoff laws: the conservation of currents at each node and the vanishing directional sum of the voltage drops around any closed loop. Let us emphasize that *R*_XY_ resistances characterize the current distributions within the circuit under consideration and cannot be determined separately. However, as will be seen below, measuring the four-probe resistance allows one to obtain a qualitative idea of the resistance of individual sections in the heterostructure studied.

**Fig. 2:**
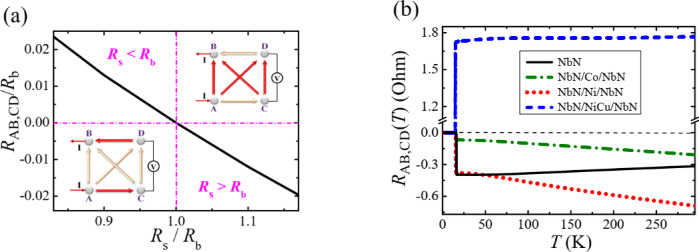
Effect of the difference between near-surface *R*_s_ and bulk *R*_b_ resistances. (**a**) Calculated four-probe resistance *R*_AB, CD_ versus the ratio *R*_s_/*R*_b_ where ${R_{{\text{AB}}}}={R_{{\text{CD}}}}={R_{{\text{AD}}}}={R_{{\text{CB}}}}={R_{\text{b}}}$ and ${R_{{\text{AC}}}}={R_{{\text{DB}}}}={R_{\text{s}}}$. The red lines in the insets indicate the dominant directions of charge flows at the surface (${R_{\text{s}}}<{R_{\text{b}}}$) and in the bulk (${R_{\text{s}}}>{R_{\text{b}}}$). (**b**) Measured temperature dependence of CPP four-probe resistances *R*_AB, CD_(*T*) for NbN(80 nm)/F(50 nm)/NbN(80 nm) trilayers with F = Co, Ni, and NiCu (dashed-dotted, dotted, and dashed lines, respectively) compared with the same characteristic for a single NbN (160 nm) layer (solid line).

To demonstrate the difference between the four-resistor schemes^24–26^ shown in Fig. 1a and the six-resistor circuit diagram in Fig. 1b, let us turn to a well-known analogy, the electrical Wheatstone bridge circuit^30^. In the simplest realization, it consists of two parallel branches linked by a third one located between intermediate points at which the difference in potentials, the circuit output, is measured. When all resistances are equal, the voltage drop across the third branch is zero due to the current balance. If all resistive components in our model coincide and are equal to *R*, then from Figs. 2a and S3, we obtain *R*_AB, CD_ = *R*_AD, CB_ = 0 in contrast to the previous model, Fig. 1a, which gives fundamentally different results ${R_{{\text{AB,}}{\kern 1pt} {\text{CD}}}}=R/4$ and ${R_{{\text{AD,}}{\kern 1pt} {\text{CB}}}}=0$, for details see Supplementary Material.

In the general case, the model shown in Fig. 1b contains too many parameters, so we assume the existence of two groups of resistances: four resistances *R*_b_, identified with the bulk (inner core) contribution, and two near-surface resistances *R*_s_. In N/F/N trilayers discussed below, the first parameter is associated with two N/F interfaces and the F film in series while the second parameter characterizes an N electrode and its interface with a Pt contact. In the case of a separate NbN layer, *R*_b_ and *R*_s_ are properties of its inner part and the near-surface area, respectively. The difference in the *R*_b_ and *R*_s_ values leads to a non-zero outcome, negative for ${R_{\text{s}}}>{R_{\text{b}}}$ and positive otherwise (Fig. 2a and Fig. S3). Moreover, small deviations of the *R*_s_/*R*_b_ ratio from unity entail relatively large variations in *R*_AB, CD_ and *R*_AD, CB_ values.

### Nonlocal four-probe resistances of N/F/N trilayers

The proposed interpretation of through-the-sample four-probe measurements was verified by changing the relationship between normal-state resistances *R*_s_ and *R*_b_ in NbN(80 nm)/F(50 nm)/NbN(80 nm) sandwiches with F = Co, Ni, and NiCu alloy (Fig. 2b). The main details of their fabrication are described in Methods. The specific resistances of the materials used can be found in the literature: *ρ*_NbN_ = 70–80 µΩ⋅cm^31,32^*ρ*_Co_ = 5.2–6.8 µΩ⋅cm^8,14,28^*ρ*_Ni_ = 3.0–6.8 µΩ⋅cm^8,33^and *ρ*_NiCu_ = 51 µΩ⋅cm^34,35^. As can be seen, the resistivities of the NbN compound and the NiCu alloy are close in value while *ρ*_Co_ and *ρ*_Ni_ are an order of magnitude smaller.

As noted above, in addition to the F-layer resistance, the S/F interfaces make their own (often dominant^12,14^) contribution to the bulk *R*_b_ resistance. It means that in NiCu-based sandwiches one should expect inequality *R*_s_ < *R*_b_, which leads to a positive value of the CPP four-probe resistance *R*_AB, CD_ according to Fig. 2a and in full agreement with the experiment (Fig. 2b). Replacing the NiCu alloy with strong ferromagnets Co or Ni, one can suppose the opposite inequality *R*_s_ > *R*_b_. Indeed, it was found^14^ that bulk N-state resistances of Nb/Co/Nb trilayers are determined primarily by the doubled Nb/Co interface contributions. Using the concerned data^14^ and the fact that the interface resistance of the contact of two dissimilar metals is roughly proportional to the sum of their resistivities^36^ we expect *R*_b_ values for NbN/Co/NbN and NbN/Ni/NbN trilayers to be much lower than in NiCu-based sandwiches and, as a result, negative *R*_AB, CD_ resistances, Fig. 2b.

In Fig. 2b, the CPP four-probe results obtained for three-layer hybrids are compared with charge transport data for a single 160 nm thick NbN film that is much thicker than its dirty-limit coherence length *ξ* of a few nm^37^. According to the paper^31^the critical temperature *T*_c_ of the N-to-S transition in NbN films is uniquely determined by the value of the product *k*_F_*l* where *l* is the electron mean free path and *k*_F_ is the Fermi wave vector. Using Fig. 1b from the work^31^the free-electron value *k*_F_ = 1.9⋅10^10^ m^− 1^, and our *T*_c_ ≈ 16 K, we find that *k*_F_*l* ≈ 8 and *l* is less than 1 nm, which is much smaller than any size in the NbN films studied. In such samples, charge transport turns into a local process that makes it probable to define a local conductivity accurately characterizing the system response to an applied electric field^38^. This statement justifies the possibility of separating the contributions of the bulk with electron transport controlled by structural defects and the scattering of electrons on random inhomogeneities at the conductor boundaries^39^.

The surface-scattering-limited regime, also known as *superdiffusive*, is qualitatively different from the ordinary bulk-scattering transport since in this case, the conductance is determined by a small fraction of itinerant electrons propagating at grazing angles with the film surface^40,41^. We speculate that just this peculiar electron transfer, which is localized in the near-surface region between two probes on the same surface, controls *R*_s_ values, while the *R*_b_ magnitudes for probes on opposite boundaries are determined by collisions with bulk defects. Although the specific intensity of the superdiffusive transfer of electrons, whose wave vectors are almost parallel to the film surface, significantly exceeds that for trajectories inside the bulk, the resistance *R*_s_ is expected to be greater than *R*_b_ due to the difference in electron paths by two orders of magnitude. This means that we are dealing with a conditional “sandwich” formed by two more resistive outer regions (NbN film surfaces) and a less resistive bulk. Immediately, following Fig. 2a, we get a negative value of *R*_AB, CD_(*T* > *T*_c_) for a single NbN layer in the normal state as follows from Fig. 2b. In general, its *R*_AB, CD_(*T*) behavior above the critical temperature *T*_c_ is сlose to a constant value since the electrical resistance of thick NbN layers changes very little with temperature, sometimes even with a negative temperature coefficient^42^.

However, with the insertion of ferromagnetic interlayers, this statement is no longer true. While *R*_AB, CD_(*T*) is almost constant for the trilayer with a NiCu film, the other two S/F/S sandwiches demonstrate a noticeable increase in the absolute value of the four-probe resistance, which remains negative. As for the NiCu alloy, the observed *R*_AB, CD_(*T*) behavior can be explained by a very low change in its resistivity over a wide temperature range, similar to NbN in the normal state^43^. As a result, the CPP four-probe resistance of NiCu-based samples does not vary noticeably with temperature. In contrast, the temperature coefficients of Ni and Co resistances of the order of 0.005–0.007 K^− 1^ belong to the highest among metals^33^which leads to an increase in *R*_b_ and, as a consequence, to the growth in the absolute value of the four-point resistance (Fig. 2b).

When the temperature decreases below *T*_c_, the resistances *R*_AB, CD_(*T* < *T*_c_) become very small. Previous studies of Nb/F/Nb trilayers^44^ showed that the doubled product of the area through which the CPP current flows and its resistance ≅ 6 × 10^− 11^ Ω сm^2^ is similar for all elemental F metals studied^8^ and is twice as high for the alloys. Using the estimates 10^− 11^ Ω сm^2^ for the interface contribution^8^ and 10^− 5^ Ω сm for the F metal resistivity we obtain the expected value of the order of 20–30 µΩ for the residual resistance of the Nb/F/Nb trilayers at *T* < < *T*_c_. Our data show a residual resistance slightly below 100 µΩ for NbN/F/NbN trilayers with F = Co and Ni and a three to four times further increase in this value for F = NiCu.

To conclude, we emphasize once again that the nonlocal implementation of the CPP four-probe technique applied to transversely inhomogeneous conducting sandwiches is capable of revealing the charge transport features hidden inside them and, thus, serving as a simple scalable diagnostic approach to characterize superconducting, spintronic, and hybrid electronic heterostructures. We expect that the developed six-resistor model will be also useful in express analyses of related non-ohmic devices with a strong dependence of transport properties on an external parameter, the role of which in the case of superconductors is played by temperature.

## Methods

### Sample preparation

Single NbN films and NbN-based trilayers on *c*-cut Al_2_O_3_ substrates were obtained by pulsed laser deposition technique in an ultrahigh vacuum chamber using an excimer KrF laser with 248 nm wavelength, the pulse duration of 35 ns, and the laser fluency of 4.94 J⋅cm^− 2^. NbN films were deposited at a constant temperature of 600 °C under 9.3 Pa pressure of the reactive N_2_ + 1% H_2_ atmosphere. For Co and Ni, the deposition of the ferromagnetic films took place at 200 °C in the Ar atmosphere under 4.5 Pa pressure and 5.2 Pa pressure for Ni_50_Cu_50_ alloy. After deposition, the chemical and structural properties of the samples were characterized by several analytical techniques^45^. Subsequently, they were patterned using optical lithography and Ar ion etching into a 25 μm × 25 μm square shape. Figure S6 in Supplementary Material shows schematically our non-magnetic/ferromagnetic/non-magnetic samples with the contact arrangement for carrying out nonlocal CPP four-probe sample measurements which, as argued in the main text of the article and Supplementary Material, strongly enhances sensitivity to inhomogeneity factors and make related experiments on hybrid trilayers the method of choice for knowing the spatial distribution of related parameters.

### Electrical measurements

The CPP nonlocal four-probe *R*_AB, CD_ (Eq. (1)) resistances were measured using the Physical Property Measurements System (PPMS) DynaCool (Quantum Design) by applying a 10 µA current into the NbN strip beneath the trilayer under study and draining it out of the Pt contact above it, see Fig. 1c in the main text and Fig. S6 in Supplementary Material. The average current density was about 1.5 A/cm^2^. In Fig. 2b, we demonstrate the non-local four-probe resistance-vs-temperature data for all-metallic hybrid sandwiches composed of two 80 nm thick NbN films and a 50 nm thick core made of three archetypal ferromagnets, Co, Ni, or NiCu alloy, which are compared with the corresponding data for single 160 nm thick NbN films.

## Electronic supplementary material

Below is the link to the electronic supplementary material.

Supplementary Material 1

## Data Availability

The datasets generated and/or analyzed during the current study are available upon request from the corresponding author. In addition, detailed information about the main characteristics of thick NbN layers can be found at https://doi.org/10.2478/jee-2019-0047 and https://doi.org/10.1016/j.apsusc.2021.149333.
