# Supplementary material for: Probing hybrid metallic sandwiches with nonlocal four-terminal electrical measurements
Source: Sci Rep. 2025 Jul 2;15:23433. doi: 10.1038/s41598-025-07507-3 (PMC12222968; doi:10.1038/s41598-025-07507-3)
Supplement: Supplementary file 1 — Supplementary Material 1 [file 41598_2025_7507_MOESM1_ESM.pdf]

Supplementary Material
for

Probing hybrid metallic sandwiches with nonlocal four-terminal electrical measurements

Mikhail Belogolovskii*, Magdaléna Poláčková, Elena Zhitlukhina, Branislav Grančič, Leonid Satrapinskyy, Pavol Ďurina, Maroš Gregor, Tomáš Plecenik

*belogolovskii@ukr.net

S1. Modeling four-probe electrical resistance measurements

Precise measurements of electrical resistances R are among the most important metrological tasks. The simplest way would be to determine it by measuring the voltage drop between two contacts that inject a specific DC current into the studied sample. Unfortunately, this approach, known as a two-point probe, is often incorrect because it involves contact resistances at the probe locations that are in series with the sample resistance. To minimize their contributions, more than a hundred years ago, an *in-line* four-probe experimental setup with two outer contacts used to supply and drain a current I from the sample and an inner pair of contacts for measuring the voltage drop V was proposed[1]. This approach provides a correct value of *local* resistance, which, however, in the general case, may have little in common with the specific electrical resistance (resistivity) ρ of the sample, the basic quantity used for the conducting material characterization. This problem can be solved exactly only in some idealized situations, in particular, for isotropic semi-infinite three-dimensional (3D) samples or infinite two-dimensional (2D) sheets[1]. In the latter case, a four-electrode *square* configuration turns out to be preferable to that along a straight line due to the smaller area and a (slightly) higher sensitivity, see Table 1 in Ref. 1.

Seventy years ago, van der Pauw (vdP)[2,3] proposed an original procedure for determining the resistivity of samples of arbitrary shape. It includes four probes located on the specimen's periphery, two contacts A and B, supplying and draining current $I = I_S = I_D$, and the other two C and D serving to determine the voltage drop V_{CD} between them. If so, then the measured *nonlocal* resistance is given by the formula

$$R_{AB,CD} = V_{CD}/I. \quad \text{(S1)}$$

Such experiments are *nonlocal* since the V_{CD} value is determined in regions far from the nominal current path and is governed by the entire current distribution across the sample. Although nonlocal measurements are usually difficult to interpret, they allow us to detect some subtle effects (in our case, the relationship between bulk and surface conductivities) that might otherwise remain unnoticed[4].

The vdP approach appears to be universal for the shape of the conductors but it has some significant limitations, namely, requires homogeneous, thin, isotropic, and singly connected samples. Resistivity measurements of anisotropic and inhomogeneous samples, which, in particular, include disordered materials, are a much more challenging problem. In this case, the main task is to limit the number of resistive parameters to be determined. For example, it is possible to obtain analytical relations for an *anisotropic* semi-infinite half plane and infinite 2D sheet measured through an in-line and square arrangement of the four probes[1]. However, even in the oversimplified situation, some artifacts, which are impossible from the conventional physical viewpoint, can emerge. First of all, it is a negative four-probe

resistance $R_{AB,CD}$ revealed, in particular, in a quasi-one-dimensional metallic surface state using an independently driven four-tip scanning tunneling microscope[5] and explained by the deformed electrostatic potential contours in the highly anisotropic sample. The application of the four-probe technique to *inhomogeneous* specimens raises even more questions. First studies[6] showed that the obtained results are sensitive to non-uniformities if their size is larger than the probe spacing and provide less information for much smaller defects. In Ref. 7, the authors proposed exact, analytic expressions for the sensitivity of four-point resistance measurements to local inhomogeneities and explained why the Hall signal can sometimes exhibit a wrong sign in n-type ZnO films. The publications mentioned above relate to normal (non-superconducting) samples. We propose and demonstrate the effectiveness of a vdP-like nonlocal approach to probe resistive characteristics of normal (N) and superconducting (S) metallic layers and related stacking heterostructures when currents are flowing perpendicular to the planes (CPP). This material supplements the main text and for the sake of completeness, repeats some of its statements.

The discussed device includes two contact pads, current and voltage (say, B and D), at the top of the sample and two contacts (say, A and C) at its bottom (see Fig. 1c in the main text). In the general case, we should follow the Landauer–Büttiker approach relating the electrical resistance to the scattering properties of the conductor and at given chemical potentials of the electrodes, determining the currents in the terminals through probabilities of electron transmissions between them[8]. In the classical domain, it is capable of introducing conditional "resistors" between the terminals R_{XY} (X, Y = A, B, C, D, and X $\neq$ Y) that describe such contributions, as is explained in the main text. After that, we can proceed with the related circuit, applying the known Kirchhoff laws: the conservation of currents at each node and the vanishing directional sum of the voltage drops around any closed loop.

In the literature, we can find two types of equivalent circuits for four-probe resistance measurements shown in Fig. S1. Each of them includes four resistors. In the first configuration, current $I_S = I_D = I$ and voltage V_{CD} are supplied and measured from opposite sides of the circuit, Fig. S1a, see, *e.g.*, Refs. 9-11. From relations $I = I_{AB} + I_{AC}$, $I_{AB}R_{AB} - I_{AC}(R_{AC} + R_{CD} + R_{DB}) = 0$ we find $V_{CD} = I_{AC}R_{CD}$ and finally the required nonlocal resistance

$$R_{AB,CD} = \frac{R_{AB} \times R_{CD}}{R_{AB} + R_{AC} + R_{CD} + R_{DB}}. \tag{S2}$$

If all resistances are identical $R_{AB} = R_{AC} = R_{CD} = R_{DB} = R$, we get $R_{AB,CD} = R/4$. In the second configuration, current $I_S = I_D = I$ and voltage V_{CB} are supplied and measured along the diagonals of the

circuit diagram, Fig. S1b, see, *e.g.*, theoretical works[12,13]. Now $I = I_{AB} + I_{AC}$, $I_{AB}(R_{AB} + R_{BD}) - I_{AC}(R_{AC} + R_{CD}) = 0$ and $V_{CB} = I_{AB}R_{AB} - I_{AC}R_{AC}$. Then the required nonlocal resistance

$$R_{AD,CB} = \frac{R_{AB} \times R_{AC} - R_{CD} \times R_{BD}}{R_{AB} + R_{AC} + R_{CD} + R_{BD}}. \quad (S3)$$

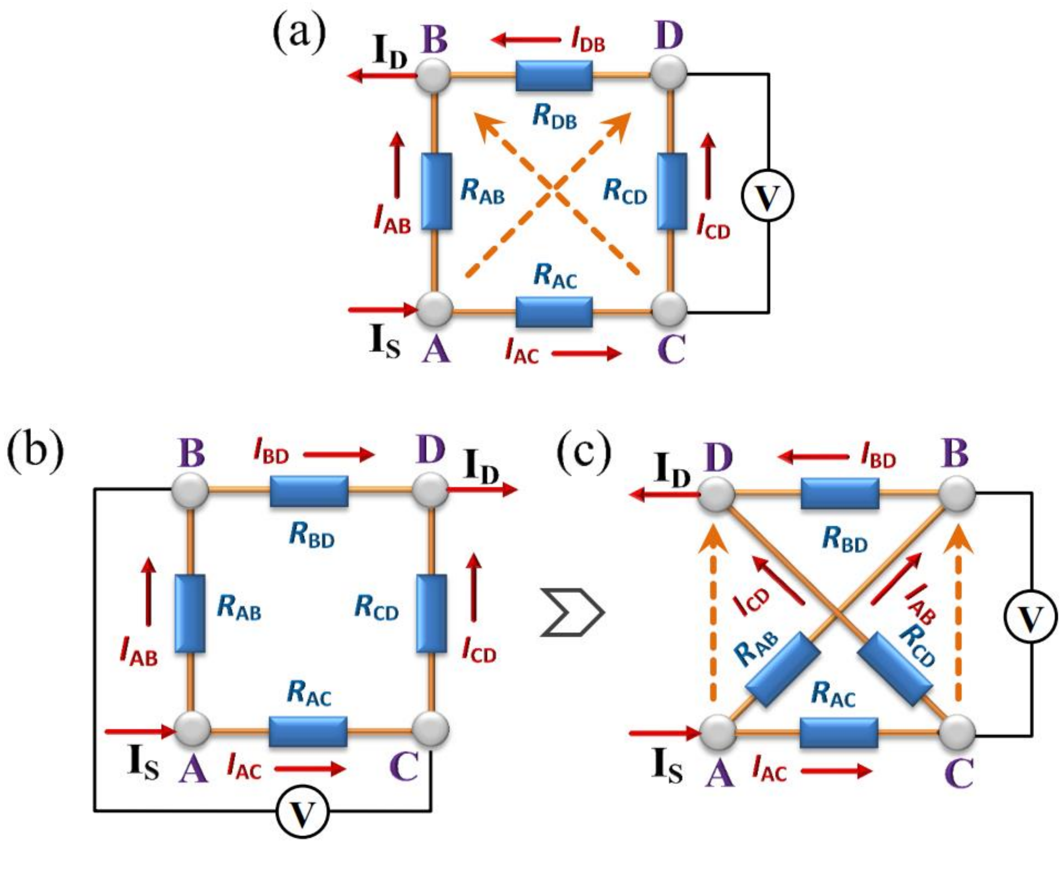

Fig. S1. Circuit diagrams explaining two types of four-resistor configurations for nonlocal four-probe electrical measurements. If, due to the charge flow redistribution, the current between the two nodes by which the voltage drop is determined changes their direction (and consequently the voltage drop changes its sign), the four-probe resistance calculated with Eq. (S1) will be negative.

Unlike the previous Eq. (S2), Eq. (S3) can lead to a formally negative value of the four-probe resistance due to the minus sign in the numerator, which is related to the fact that the voltage drop between nodes B and C consists of two oppositely directed components V_{AB} and V_{AC}. As a result, its sign, as well as the sign of the four-probe resistance $R_{AD,CB}$, can be negative dependently on the values of the individual resistances. Moreover, for identical resistances $R_{AB} = R_{AC} = R_{CD} = R_{BD} = R$, we get $R_{AD,CB} = 0$. The difference between the two approaches is clearly visible when replacing node B with D and vice versa (Fig. S1c). It results in

the same configuration as in Fig. S1a, but now with two diagonal links between the nodes and two side ones instead of four in Fig. S1a.

Since the Landauer–Büttiker approach[8] relating scattering characteristics of electrons in a device to its conducting properties deals with the transmission probabilities between *each* two nodes, it is natural to expand the two above schemes by including the missing links shown conditionally by dashed arrows in Fig. S1. Such a generalized circuit with six resistors instead of four is proposed in the main text in Fig. 1b. It is easy to see that the configuration of our samples (Fig. 1c) corresponds exactly to the case when the transmission probabilities between terminals A and B, as well as between A and D, are approximately the same, thus, ignoring diagonal connections would be incorrect.

S2. The six-resistor model for four-probe electrical measurements

Below we demonstrate our circuit diagram with six resistors that, as we argue, more adequately describes *through-sample* CPP resistance measurements (Fig. S2).

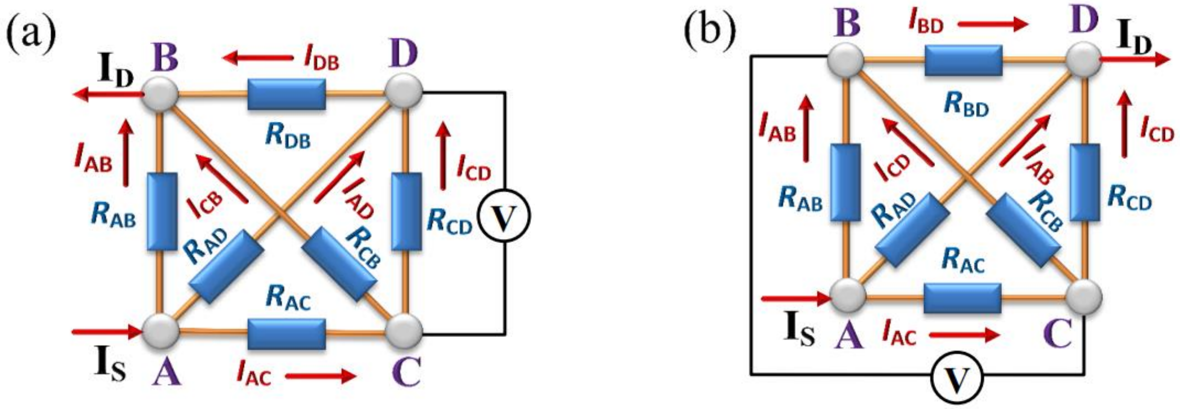

Fig. S2. Circuit diagram with six conditional resistances for a comprehensive description of $R_{AB,CD}$ and $R_{AD,CB}$ results obtained by non-local four-probe through-sample electrical measurements. Resistances R_{DB} (or R_{BD}) and R_{AC} describe the near-surface transport between contacts on the same surface of the sample studied while four other resistances characterize the bulk transport between contacts on its opposite surfaces.

Calculations of the four-probe resistance $R_{AB,CD}$ is now based on a larger number of equations:

$$I = I_{AB} + I_{AC} + I_{AD}; \quad I_{AC} = I_{CB} + I_{CD}; \quad I_{AB}R_{AB} - I_{CB}R_{CB} - I_{AC}R_{AC} = 0; \quad I_{AB}R_{AB} - I_{DB}R_{DB} - I_{AD}R_{AD} = 0;$$

$$I_{CB}R_{CB} - I_{DB}R_{DB} - I_{CD}R_{CD} = 0; \quad I_{AB}R_{AB} - I_{DB}R_{DB} - I_{CD}R_{CD} - I_{AC}R_{AC} = 0; \quad V_{CD} = I_{CD}R_{CD} \qquad \text{(S4)}$$

This system of linear equations can be solved numerically or analytically. Since the final analytical expression is very cumbersome, we do not give it here. The results obtained, which depend on the mutual

relationships between the resistance values included in the circuit, are now much more diverse compared to the two configurations discussed above. Fig. 2a in the main text of the paper shows how the sign of $R_{AB,CD}$ changes with varying the ratio between the resistances $R_{DB} = R_{AC} = R_s$ describing the near-surface transport between contacts on the same surface of the sample and other resistances characterizing the bulk transport between contacts on its opposite surfaces. Note also that the introduction of diagonal links into the circuit diagram shown in Fig. S1a and side links into the circuit diagram shown in Fig. S1c allows phenomenologically explaining the appearance of the negative four-probe resistance in the calculations where the current *through* a device depends significantly on the coordinates[14-16]. Indeed, the model we propose is in no way limited to a specific implementation of the two types of conditional resistances, which we call surface R_s and bulk R_b. It can be, *e.g.*, a tunnel junction with two metal plates separated by an insulating layer in which negative four-probe resistance occurs when the film resistance exceeds the barrier resistance[14], *i.e.*, $R_s > R_b$, see Fig. S3. A similar example is the contact of two wires with a device whose resistance is lower than that of the wires[15] or complex interfaces with comparatively low resistances formed by high-T_c superconducting and ferromagnetic thin films[16].

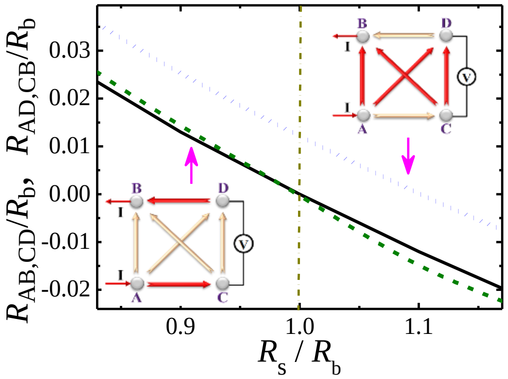

Fig. S3. Calculated four-probe resistances $R_{AB,CD}$ and $R_{AD,CB}$ versus the R_s/R_b ratio where $R_{AB} = R_{CD} = R_b$, $R_{AD} = R_{CB} = R_d$ and $R_{AC} = R_{DB} = R_s$. When $R_d = R_b$, both curves $R_{AB,CD}/R_b$-vs-R_s/R_b and $R_{AD,CB}/R_b$-vs-R_s/R_b coincide (solid line). Dotted and dashed lines correspond to $R_{AD,CB}/R_b$-vs-R_s/R_b and $R_{AB,CD}/R_b$-vs-R_s/R_b characteristics for $R_d = 1.1R_b$. The red lines in the insets indicate dominating directions of charge flows at the surface ($R_s < R_b$) and in the bulk ($R_s > R_b$).

As argued in the main text, the dependence of four-point resistances $R_{AB,CD}$ and $R_{AD,CB}$ on the values of the resistive components is similar to that for the Wheatstone bridge. If all resistances are the same, the result is exactly zero for both configurations. In the case where the surface resistances differ from the bulk ones, these values can be positive or negative depending on the ratio R_s/R_b. In Fig. S3, we show what

further breaking of the circuit symmetry leads to. For example, when the diagonal resistances $R_{AD} = R_{CB} = R_d$ slightly exceed the R_b value, $R_{AB,CD}$ deviates from $R_{AD,CB}$ while the general tendency to change the sign near $R_s = R_b$ remains the same (Fig. S3).

Even more unusual is the four-probe resistance for a superconducting sample in the temperature interval that corresponds to the normal-to-superconducting state transition, *i.e.*, a drop in resistance from a finite value to zero. The behavior of all resistances which may have slightly different superconducting parameters are approximated by identical formulas $R(T) = R^*\left(1+\tanh\left((T - T_c)/\delta T_c\right)\right)$ where R^* corresponds to the *middle point* of the resistive transition (in experiments, it is often determined as a value between 10 and 90% *resistivity* drop) and δT_c determines its width.

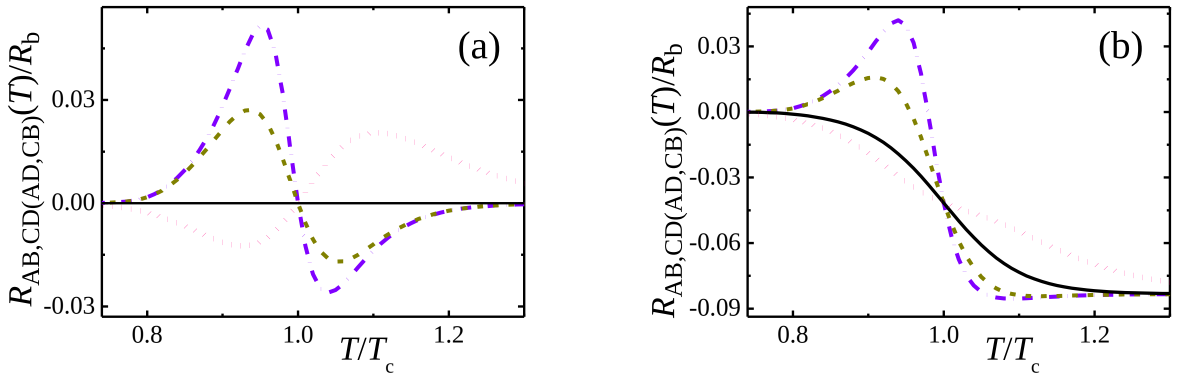

Fig. S4. Calculated temperature dependences of four-probe resistances $R_{AB,CD}$ (T) = $R_{AD,CB}$ (T), $T_{c,AB}$ = $T_{c,AD}$ = $T_{c,CB}$ = $T_{c,CD}$ = $T_{c,AC}$ = $T_{c,DB}$ = $T_{c,b}$, $\delta T_{c,AB}$ = $\delta T_{c,AD}$ = $\delta T_{c,CB}$ = $\delta T_{c,CD}$ = $0.1T_{c,b}$, $\delta T_{c,AC}$ = $\delta T_{c,DB}$ = $0.03T_{c,b}$, $0.05T_{c,b}$, $0.1T_{c,b}$, $0.2T_{c,b}$ (dashed-dotted, dashed, solid, and dotted curves, respectively): (*a*) R_{AB} = R_{AD} = R_{CB} = R_{CD} = R_{AC} = R_{DB} = R_b; (*b*) R_{AB} = R_{AD} = R_{CB} = R_{CD} = R_b, R_{AC} = R_{DB} = $2R_b$.

When all normal and superconducting factors coincide, the output four-probe resistance tends to zero (solid curve in Fig. S4a). The difference in normal-state resistances leads to the usual resistance-versus-temperature dependence at the N-to-S transition, which under certain conditions can be inverted upside down (solid curve in Fig. S4b). Fig. S4 shows that even small changes in superconducting parameters, such as the transition width δT_c, are clearly visible against the background in nonlocal four-probe measurements.

Fig. S5 demonstrates the fundamental difference between the results for $R_{AB,CD}(T)$ and $R_{AD,CB}(T)$ arrangements arising when the superconducting parameters for the diagonal links AD and CB differ from the others while the difference between the normal resistances is the same as in Fig. S4b.

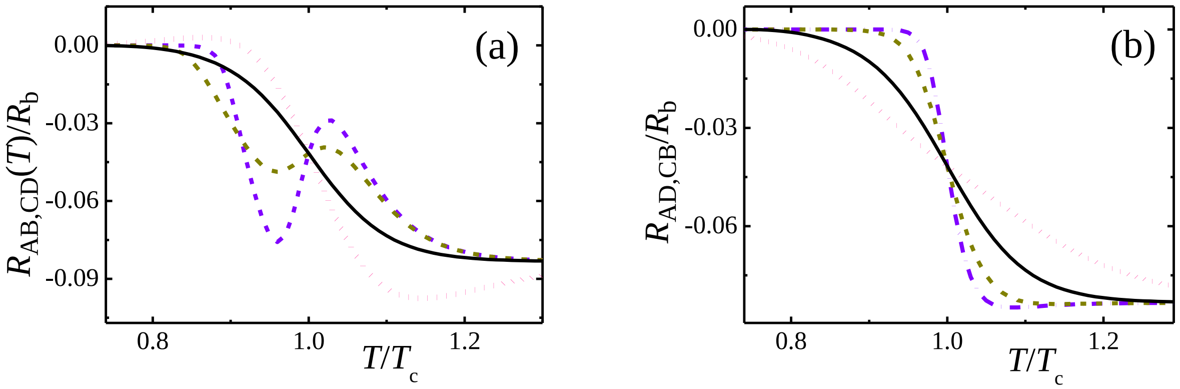

Fig. S5. Calculated temperature dependences of four-probe resistances $R_{AB,CD}$ (T) (a) and $R_{AD,CB}$ (T) (b), R_{AB} = R_{AD} = R_{CB} = R_{CD} = R_b, R_{AC} = R_{DB} = 2R_b, $T_{c,AB}$ = $T_{c,AD}$ = $T_{c,CB}$ = $T_{c,CD}$ = $T_{c,b}$, $T_{c,AC}$ = $T_{c,DB}$ = $T_{c,b}$, 0.95$T_{c,b}$, 0.9$T_{c,b}$ (dashed-dotted, dashed, and dotted curves in both figures), $\delta T_{c,AB} = \delta T_{c,AD} = \delta T_{c,CB} = \delta T_{c,CD}$ = 0.1$T_{c,b}$, $\delta T_{c,AC} = \delta T_{c,DB}$ =0.03$T_{c,b}$. Black solid lines correspond to *uniform* superconducting properties.

S3. Schematic representation of the N/F/N CPP configuration for nonlocal four-probe electrical measurements

Our non-magnetic/ferromagnetic/non-magnetic (N/F/N) sample with related four probes A, B, C, and D, as well as biasing contacts, is shown below in two schematic versions: a 3D configuration and a simplified 2D illustration of its basic features.

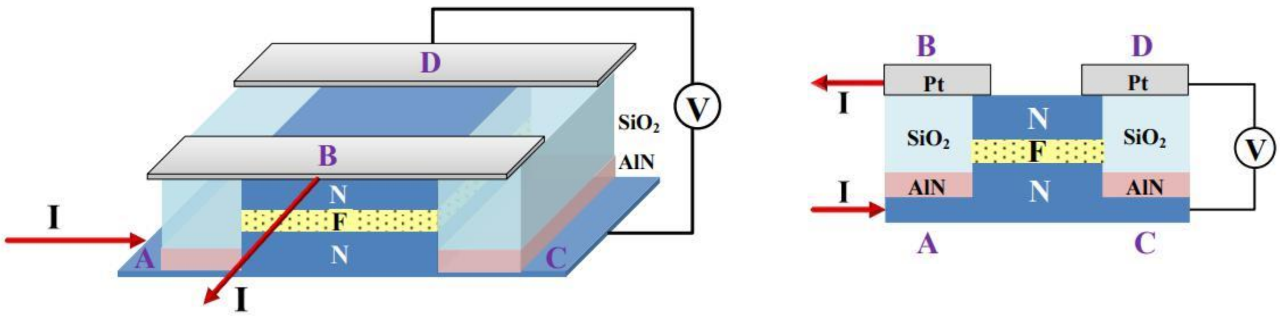

Fig. S6. Schematics of an N/F/N sandwich formed by non-magnetic (N) and ferromagnetic (F) metal layers with the measurement biasing for probing the CPP nonlocal four-terminal resistance $R_{AB,\,CD} = V_{CD}/I_{AB}$: a 3D representation (left) and a simplified 2D illustration (right). The current input and output were carried out through terminals A and B, respectively; the voltage drop V_{CD} was measured between the C and D terminals. The samples were protected by side bilayers formed by a 30 nm thick AlN layer and about 200 nm thick SiO_2 oxide.

References

1. I. Miccoli, F. Edler, H. Pfnur, and C. Tegenkamp, "The 100th anniversary of the four-point probe technique: the role of probe geometries in isotropic and anisotropic systems," *J. Phys.: Condens. Matter*. **27**, 223201 (2015). https://doi.org/10.1088/0953-8984/27/22/223201
2. L. J. van der Pauw, "A method of measuring specific resistivity and Hall effect of discs of arbitrary shape," *Philips Res. Rep.* **13**, 1 (1958).
3. L. J. van der Pauw, "A method of measuring the resistivity and Hall coefficient on lamellae of arbitrary shape," *Philips Tech. Rev.* **20**, 220 (1958).
4. D. A. Abanin, S. V. Morozov, L. A. Ponomarenko, R. V. Gorbachev, A. S. Mayorov, M. I. Katsnelson, K. Watanabe, T. Taniguchi, K. S. Novoselov, L. S. Levitov, A. K. Geim, "Giant nonlocality near the Dirac point in graphene," *Science* **332**, 328 (2011). https://doi.org/10.1126/science.1199595
5. T. Kanagawa, R. Hobara, I. Matsuda, T. Tanikawa, A. Natori, and S. Hasegawa, "Anisotropy in conductance of a quasi-one-dimensional metallic surface state measured by a square micro-four-point probe method," *Phys. Rev. Lett.* **91**, 036805 (2003). https://doi.org/10.1103/PhysRevLett.91.036805
6. L. J. Swartzendruber, "Four-point probe measurement of non-uniformities in semiconductor sheet resistivity," *Solid-State Electron.* **7**, 413 (1964). https://doi.org/10.1016/0038-1101(64)90038-3
7. D. W. Koon, F. Wang, D. H. Petersen, and O. Hanse, "Sensitivity of resistive and Hall measurements to local inhomogeneities," *J. Appl. Phys.* **114**, 163710 (2013). https://doi.org/10.1063/1.4826490
8. D. A. Ryndyk, "Landauer-Büttiker method," in: *Theory of Quantum Transport at Nanoscale*. Springer Series in Solid-State Sciences (Springer, Cham, 2016). Vol 184. https://doi.org/10.1007/978-3-319-24088-6_2
9. D. W. Koon and C. J. Knickerbocker, "What do you measure when you measure resistivity?" *Rev. Sci. Instrum.* **63**, 207 (1992). https://doi.org/10.1063/1.1142958
10. R. Vaglio, C. Attanasio, L. Maritato, A. Ruosi, "Explanation of the resistance-peak anomaly in nonhomogeneous superconductors," *Phys. Rev. B* **47**, 15302 (1993). https://doi.org/10.1103/PhysRevB.47.15302
11. M. Poláčková, E. Zhitlukhina, M. Belogolovskii, M. Gregor, T. Plecenik, and P. Seidel, "Probing superconducting granularity using nonlocal four-probe measurements," *Eur. Phys. J. Plus* **138**, 486 (2023). https://doi.org/10.1140/epjp/s13360-023-04123-w
12. H. B. G. Casimir, "On Onsager's principle of microscopic reversibility," *Rev. Mod. Phys.* **17**, 343 (1945). https://doi.org/10.1103/RevModPhys.17.343
13. M. Büttiker, "Symmetry of electrical conduction," *IBM J. Res. Develop*. **32**, 317 (1988). https://doi.org/10.1147/rd.323.0317.
14. R. J. Pedersen and F. L. Vernon, Jr, "Effect of film resistance on low-impedance tunneling measurements," *Appl. Phys. Lett.* **10**, 29 (1967). https://doi.org/10.1063/1.1754793
15. J. M. Pomeroy and H. Grube, " 'Negative resistance' errors in four-point measurements of tunnel junctions and other crossed-wire devices," *J. Appl. Phys.* **105**, 094503 (2009). https://doi.org/10.1063/1.3122503
16. T. Nurgaliev, V. Štrbík, N. Gál, Š. Chromik, and M. Sojková, "Electrical transport effects in YBCO/LSMO bilayer junctions," *Physica B: Condens. Matter.* **550**, 324 (2018). https://doi.org/10.1016/j.physb.2018.09.021
